# Supplementary material for: An iPSC-derived neuronal model reveals manganese’s role in neuronal endocytosis, calcium flux and mitochondrial bioenergetics
Source: iScience. 2025 Aug 6;28(9):113311. doi: 10.1016/j.isci.2025.113311 (PMC12410566; doi:10.1016/j.isci.2025.113311)
Supplement: Document S1. Figures S1–S9 and Tables S1, S3, and S4 [file mmc1.pdf]

iScience, Volume *28*

Supplemental information

An iPSC-derived neuronal model reveals manganese's role in neuronal endocytosis, calcium flux and mitochondrial bioenergetics

Dimitri Budinger, Sharmin Alhaque, Ramón González-Méndez, Chris Dadswell, Katy Barwick, Arianna Ferrini, Charlotte Roth, Conor J. McCann, Karin Tuschl, Fatma Al Jasmi, Maha S. Zaki, Julien H. Park, Russell C. Dale, Shekeeb Mohammad, John Christodoulou, Dale Moulding, Michael R. Duchen, Serena Barral, and Manju A. Kurian

Table S1. Summary for the clinical information of patients used in this study, related to Figure 1.

	SLC39A14		SLC39A8		SLC30A10	
	Patient 1	Patient 2	Patient 1	Patient 2	Patient 1	Patient 2
**Mutation**	c.[1407C>G]	c.[781-9C>G]	c.[338G>C]	c.[112G>C], c.[1019T>C]	c.[314_322del]	c.[77T>C]
**Amino acid change**	p.[N469K]	p.[H251Pfs26]	p.[C113S]	p.[G38R], p.[I340N]	p.[A105_P107del]	p.[L26P]
**Homozygous/Heterozygous (HO/HET)**	HO	HO	HO	Compound HET	HO	HO
**Predicted effect**	Missense	Splice-site	Missense	Missense	Inframe deletion	Missense
	**Patients characteristics**					
**Sex**	Female	Female	Female	Female	Female	Female
**Consanguinity**	Y	Y	Y	N	Y	Y
**Age of onset (average)**	2 years	22 months	3 months	4 months	11 years	2.5 years
	**Patients clinical phenotypes**					
**Initial symptoms**	Abnormality in gait, postural dystonia, toe walking, plantar flexion	Delayed motor development,	Developmental delay, flopiness, dystonia, skeletal abnormalities	Infantile spasms, cranial synostosis, dysproportionale short stature	Gait disturbance, dystonia	Gradual motor regression
**Subsequent symptoms**	Generalized dystonia, oromandibular dystonia, limb contracture, scoliosisacquired microcephaly	Progressive generalized dystonia, loss of ambulation	Limbs stiffening, broad forehead, scoliosis, seizures, hypotonia	hepatopathy, spasticity	Liver cirrhosis, generalized dystonia, dysarthria, bradykinesia	Generalized dystonia, hypomimia, rigidity, bradykinesia
**Whole-blood Mn (73-325 nmol/L) (average)**	2457	1,168	<0.1	<0.1	3285	3038
**Response to treatment**	No improvements following chelation therapy ($Na_2CaEDTA$)	Slight improvement of motor function following chelation therapy ($Na_2CaEDTA$)	Patient improved with Mn supplementation	Patient improved with Mn supplementation	Good response, with improved motor functions following chelation therapy ($Na_2CaEDTA$)	Fair to good motor improvement following chelation therapy ($Na_2CaEDTA$)
**MRI findings**	T1-hyperintensity of GP, striatum, PG, DP, cerebellum	T1-hyperintensity GP and SN	Cerebral atrophy, T2-hyperintensity of BG, putamen, GP	Cerbral and cerebellar atrophy, T2-hyperintensity of BG, putamen, GP	T1-hyperintensity of CN, P, GP, SN, DN, AP, WM	T1 hyperintensity of BG, BS, CB
**Reference**	Tuschl et al., 2016	Rodan et al., 2018	Riley *et al*., 2017	Park et al., 2015, 2018	Tuschl et al., 2008, 2012	Zaki et al., 2018

GP, globus pallidus; PG, pituitary gland; DP, dorsal pons; SN, substantia nigra; BG, basal ganglia; CN, caudate nucleus; P, putamen; DN, dental nucleus; AP, anterior pituitary; WM, white matter; BS, brainstem

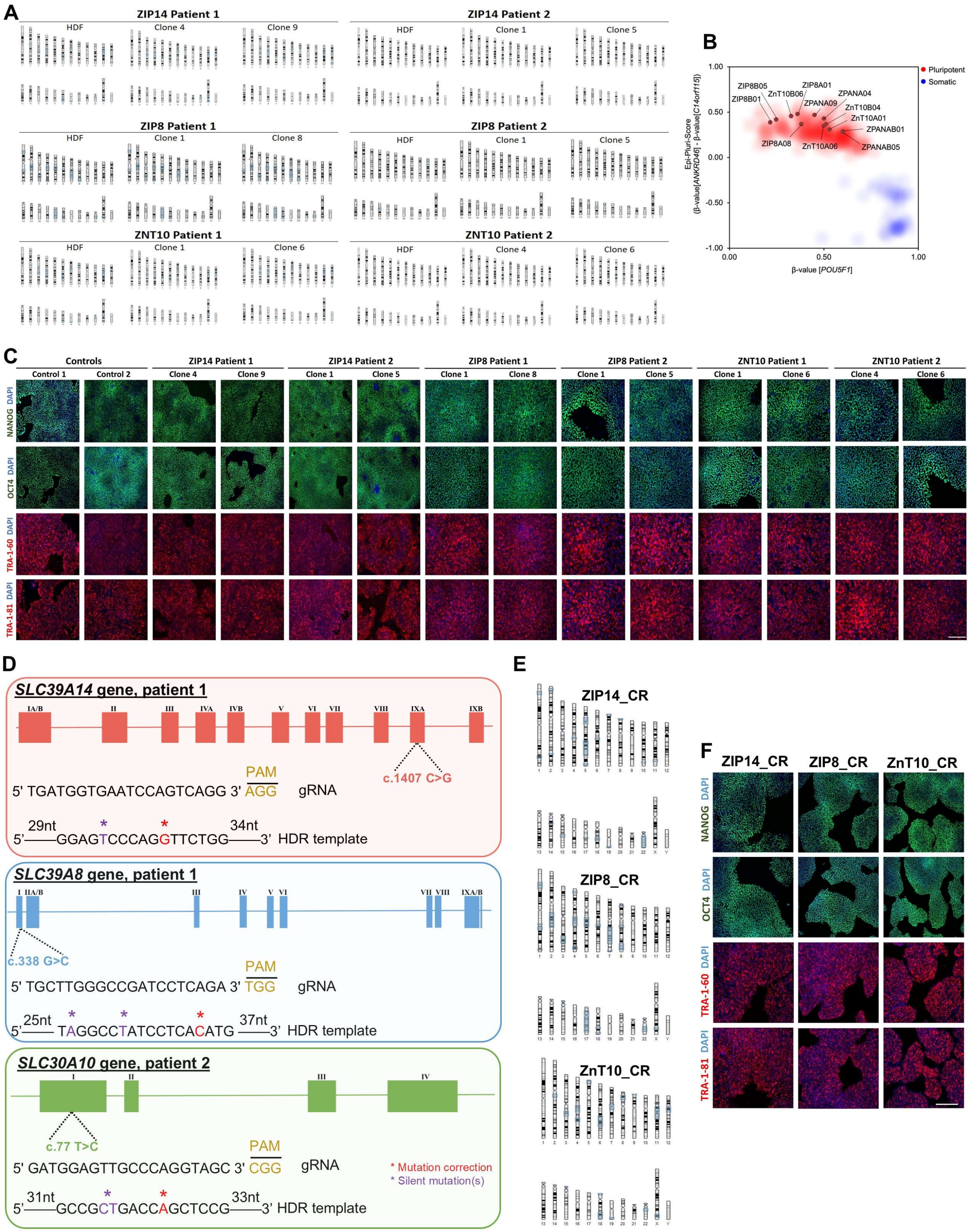

Figure S1. iPSC characterization and generation of CRISPR-Cas9 isogenic controls, related to Figure 1.

(A) SNP analyses of the iPSC lines generated in this study show normal karyotype, without significant differences to derived fibroblast line. Two clones per line were initially characterized and compared to the original human dermal fibroblast (HDF).

(B) Epi-Pluri-Score analysis of iPSC lines show all generated lines cluster within the pluripotent stem cell lines (red), rather than with somatic cell lines (blue). Analysis generated by Cygenia, Epigenetic Diagnostics, Aachen, German.

(C) Immunofluorescence analysis for pluripotency markers OCT4, NANOG, TRA-1-60, and TRA-1-81 show all iPSC lines express these pluripotent markers. Scale bar, 100 µm.

(D) gRNA and HDR repair template design for CRISPR-Cas9 single nucleotide correction in the *SLC39A14* patient 1, *SLC39A8* patient 1, and *SLC30A10* patient 2 iPSC lines.

(E) SNP analyses of the CRISPR-corrected iPSC lines (ZIP14 CR02, ZIP8 CR04, and ZnT10 CR04) show normal karyotype, without significant differences to derived fibroblast line.

(F) Immunofluorescence analysis for pluripotency markers OCT4, NANOG, TRA-1-60, and TRA-1-81 show all isogenic control lines express these pluripotent markers. Scale bar, 100 µm.

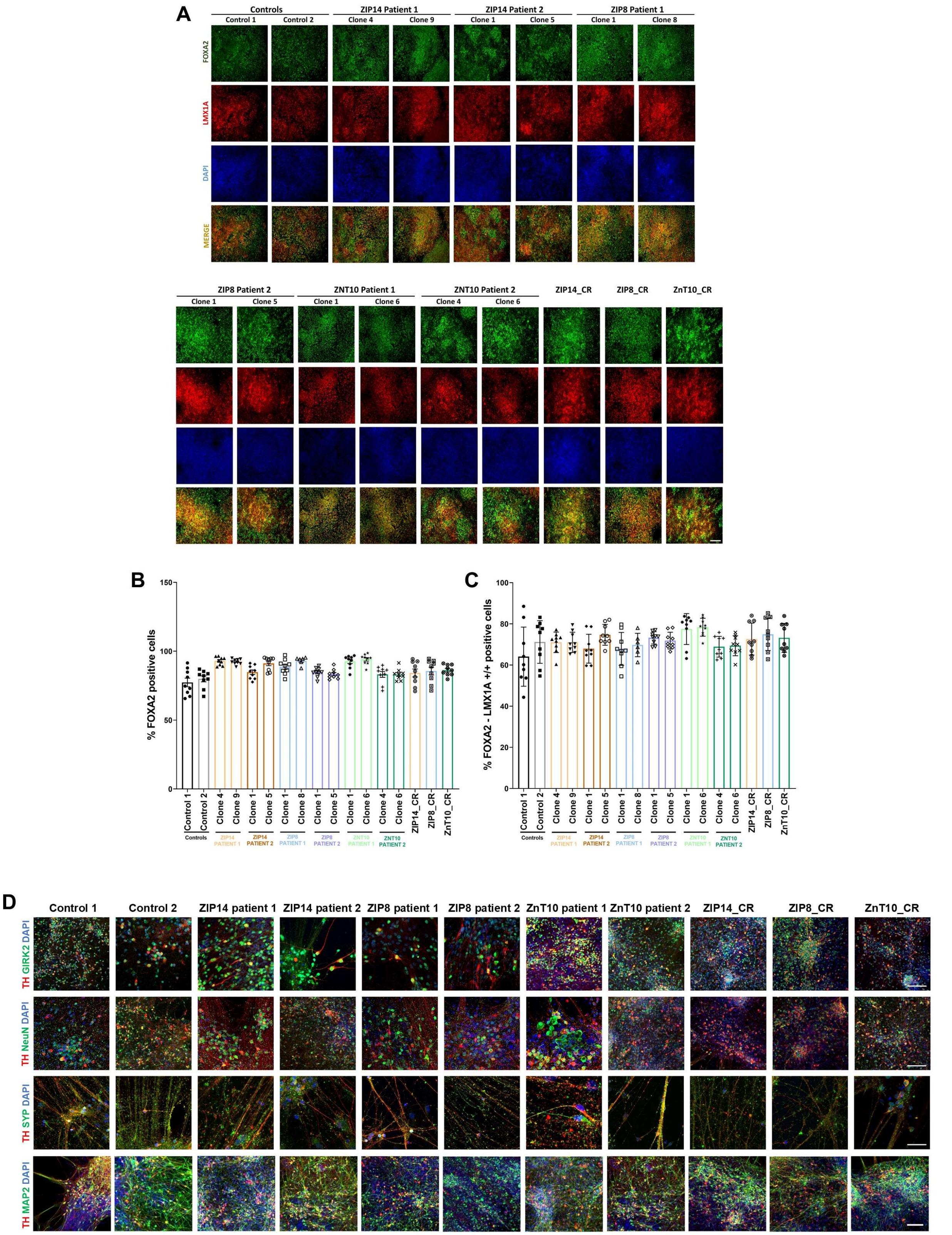

Figure S2. Characterization of iPSC-derived mDA neuronal identity at precursor (day 11) and mature stage (day 65), related to Figure 1

(A) Images of day 11 neuron precursors stained for midbrain progenitor markers FOXA2 and LMX1A.

(B-C) Quantitative analysis of DAPI/FOXA2 positive cells at day 11 of mDA differentiation (B) and quantitative analysis of FOXA2/LMX1A positive cells at day 11 of mDA differentiation (C). Comparable to the literature, there is a high percentage of cells double-positive for FOXA2/LMX1A, suggesting an acceptable level of mDA neuronal progenitors. Values are given as means ± SEM.

(D) Immunofluorescence analyses for neuronal maturity markers GIRK2, NeuN, SYP, and MAP2 together with mDA marker TH. Here are shown selected clones for each patient lines that were carried forward for downstream experiments, ZIP14 patient 1 clone 9, ZIP14 patient 2 clone 1, ZIP8 patient 1 clone 1, ZIP8 patient 2 clone 1, ZnT10 patient 1 clone 1, and ZnT10 patient 2 clone 6 were selected. Scale bar, 100 µm.

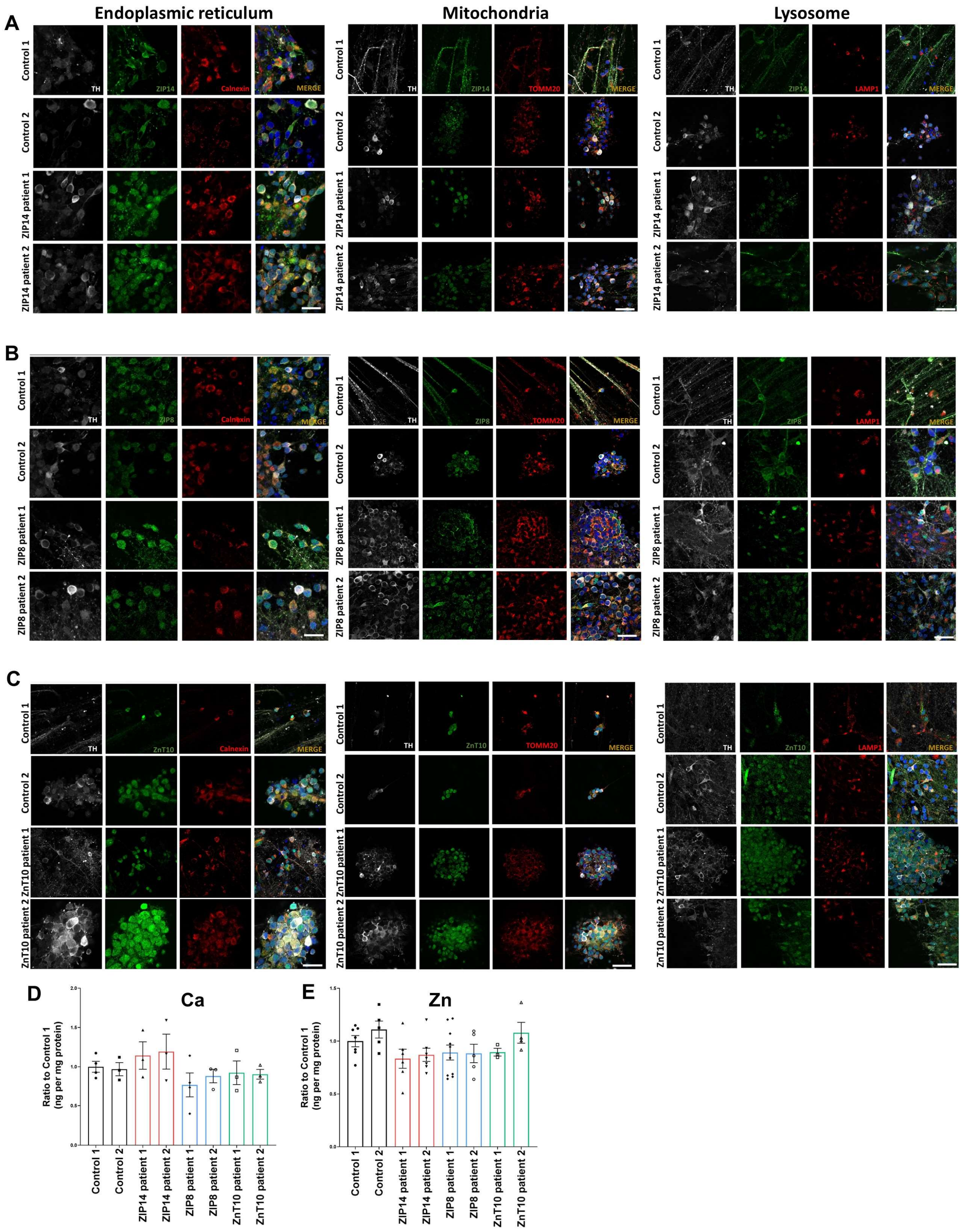

Figure S3. Subcellular localization of metal-ion transporters ZIP14, ZIP8, and ZnT10 in mDA neurons and Ca and Zn intracellular levels, related to Figure 1.

(A-C) Immunofluorescence for the endoplasmic reticulum (calnexin), mitochondria (TOMM20), and lysosomes (LAMP1) in TH-positive neurons show co-localization of ZIP14, ZIP8, and ZnT10 with the ER, mitochondria, and nucleus (DAPI), but not with lysosomes. Scale bars, 20 µm (ER) and 50 µm (TOMM20, LAMP1).

(D-E) ICP-MS analysis for intracellular levels of calcium (D) and zinc (E) do not show significant intracellular differences. Unpaired Student's t test. Values are given as means ± SEM.

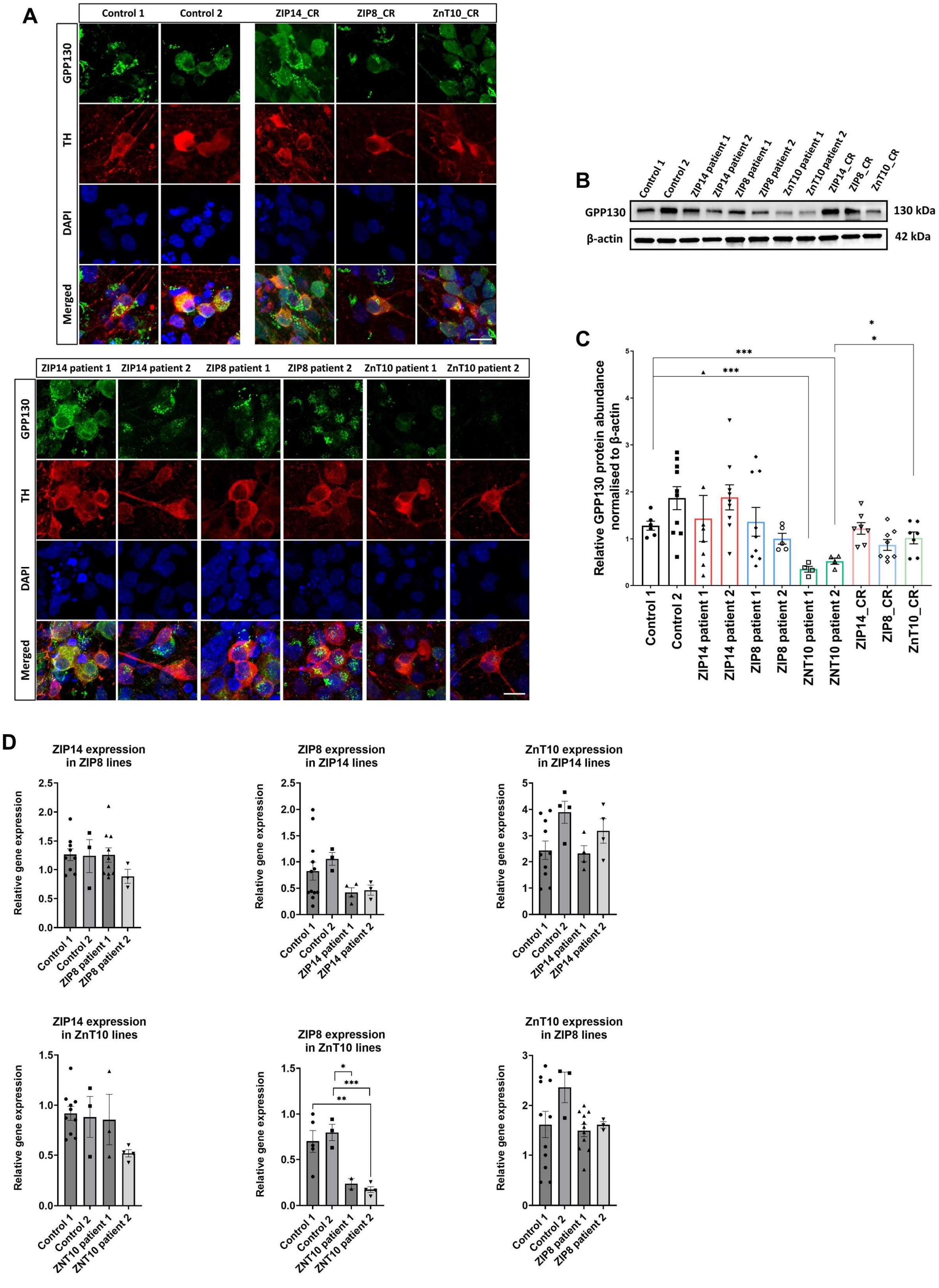

Figure S4. Immunofluorescence for the manganese-specific sensor GPP130 for the efflux activity of ZnT10 transporter, and transporters relative gene expression, related to Figure 1.

(A) Day 65 neurons were stained for the Golgi marker GPP130 and mDA marker TH, which show a reduction in GPP130 signal intensity in the ZnT10 patient lines. Scale bar, 10 µm.

(B-C) Immunoblot of total GPP130 protein (130 kDa) with β-actin (45 kDa) as housekeeping gene (B). Relative abundance of GPP130 protein, normalized to β-actin (C). n= 4 – 10 biological replicates, unpaired Student's t test; *p=0.05-0.01, **p=0.01-0.001, p***< 0.001. Values are given as means ± SEM.

(D) Effect of mutations on gene expression of the other two Mn transporters by qRT-PCR analyses for ZIP14, ZIP8, and ZnT10 relative to GAPDH and normalized to an internal control in the different patient lines. n= 3 – 12 biological replicates, unpaired Student's t test; *p=0.05-0.01, **p=0.01-0.001, p***< 0.001. Values are given as means ± SEM.

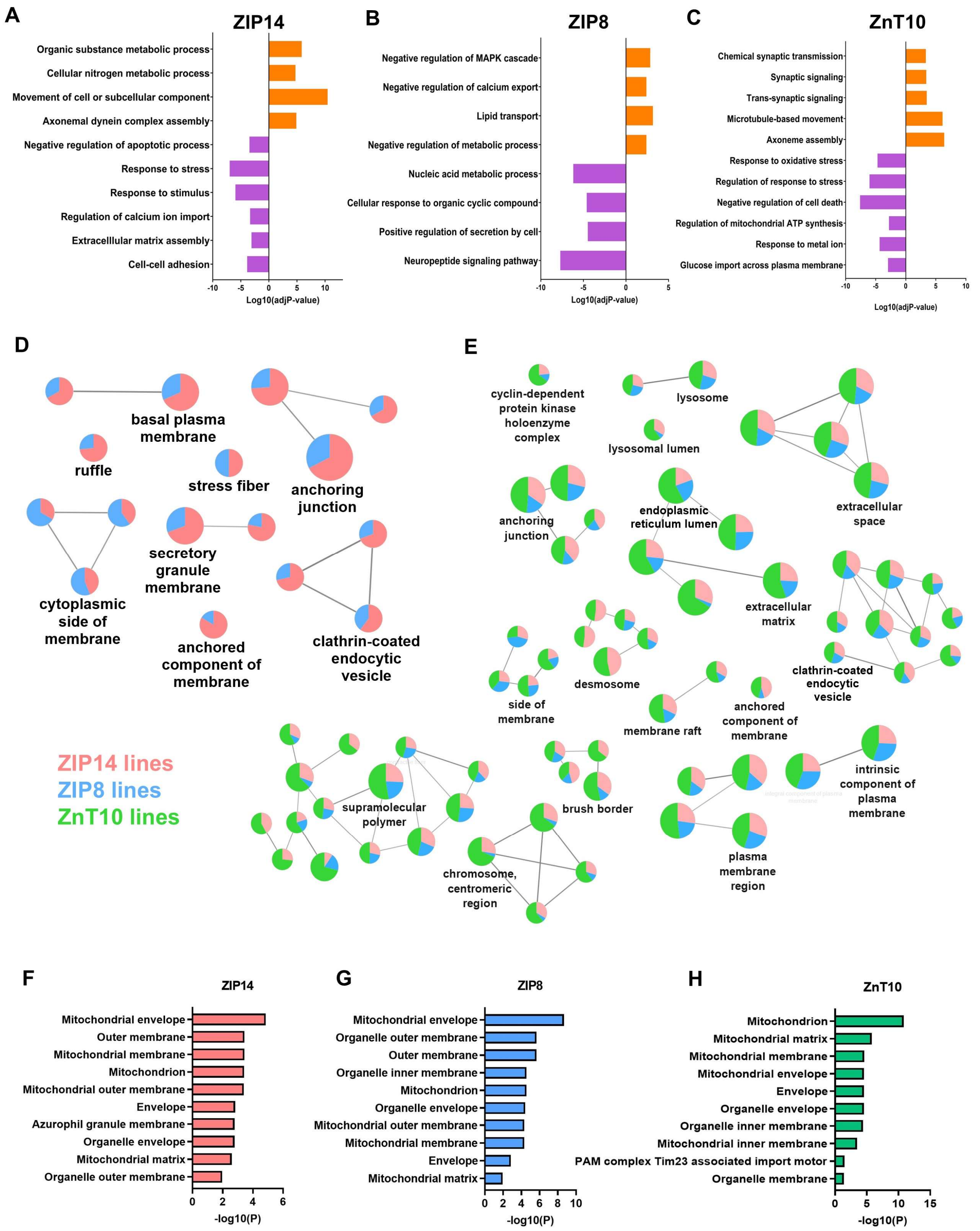

Figure S5. Dysregulation of biological processes and cellular components in ZIP14, ZIP8, and ZnT10 lines, related to Figure 2 and Table S2-S3.

(A-C) Gene ontology (GO) terms enrichment for biological process of underexpressed (blue) and overexpressed (red) protein coding genes in ZIP14 (A), ZIP8 (B), and ZnT10 (C) lines.

(D-E) ClueGOterms enrichment for cellular components dysregulated in both ZIP14 and ZIP8 patient lines (D) or between all three disorders (E). Network graph nodes represent GO terms and node size correlates with the number of genes participating in that pathway, edge (connections) indicate shared genes between GO terms. Only the GO functional groups exhibiting higher statistically significant differences, using Benjamini-Hochberg P-value correction (FDR<0.05) are shown.

(F-H) Gene ontology (GO) terms enrichment for cellular process dysregulated in ZIP14 (F), ZIP8 (G), and ZnT10 (H) patient lines. These terms were highlighted by cross-referencing DEGs with the MitoCarta 3.0 database and enrichment was performed using ShinyGO 0.76.2.

Table S3. Shared DEGs associated with SLC39A14, SLC39A8, and SLC30A10 disorders using PathCards, related to Figure 2.

Condition	Diseases of glycosylation: Downregulated	Diseases of glycosylation: Upregulated	Extracellular matrix organisation: Downregulated	Extracellular matrix organisation: Upregulated	Vesicle-mediated transport, including endocytosis: Downregulated	Vesicle-mediated transport, including endocytosis: Upregulated
ZIP14 basal condition	ADAMTS6, MUC15, ADAMTSL4, ACAN, ADAMTS18, FMOD, CSPG4, ADAMTS9, ADAMTSL2, CYP11A1, B4GALT1, PPP1R3C	SEMA5B, LINC01238, CUBN	NID2, COL15A1, CTSV, CAPN6, CDH1, KDR, ITGA5, ITGA8, ACAN, LAMC2, ADAMTS18, LAMB1, MFAP4, FMOD, SCUBE3, COL1A2, ADAM12, F11R, ADAMTS9, LAMB3, NID1, SPP1	COL28A1, SCUBE1, LINC01238, COL4A3, COL27A1, COL4A4	VAMP8, AP1M2, APOE, CTSZ, FOLR1, EGF, KIF2C, CD4, COL1A2, KDELR3, SYTL1, RINL, CD55, SSC5D	LINC01238, F5
ZIP8 basal condition	MUC5AC, MUC15, ADAMTS18, SBSPON, ADAMTSL3, MUC5B, CYP11A1, PPP1R3C, MAOA	None	SCUBE3, COL23A1, FURIN, ITGB8	ICAM5	CTSZ, CHMP4C, TACR1, SH3KBP1, CHRM2, KIF6, SPTB	SLC18A3
ZnT10 basal condition	THBS1, MUC5AC, FMOD, LUM, ADAMTSL4, ADAMTS1, ADAMTS18, ADAMTS6, CSPG4, SPON2, HSPG2, NOTCH3, ADAMTS9, THBS2, CYP1B1, B4GALT1, C1GALT1C1, PPP1R3C, CYP11A1, CYP26B1, SDC1	ADAMTS5, SFTPC	COL15A1, COL6A3, KDR, CDH1, THBS1, NID2, CTSV, ITGA5, COL12A1, COL1A1, COL17A1, ITGA8, FN1, FMOD, COL14A1, LUM, COL3A1, LOXL2, SPP1, LAMB1, ADAMTS1, COL1A2, LAMC2, COL5A1, ADAMTS18, COL4A1, FBN2, P3H2, COL21A1, EMILIN1, MMP2, MMP14, VTN, NID1, LAMB3, ELN, FBLN1, F11R, SCUBE3, SERPINH1, COL5A2, ADAM12, HSPG2, COL13A1, SERPINE1, FBLN2, ADAMTS9, LOX, LAMC1, SDC1, ADAM9, ICAM1	ADAMTS5	SERPINA1, COL1A1, VAMP8, COL3A1, APOE, KIF20A, COL1A2, KIF23, KIF2C, COL4A1, CTSZ, KIFC1, LRP2, KIF11, KDELR3, CTSC, CD4, TBC1D8B, MASP1, SYNJ2, CHMP4C, TRIP10, KIF15, KIF4A, CD55	TUBA1C, SLC18A3
Controls treated	SDC1, PRELP	SPON1, GPC3	EMILIN2, SDC1	BMP1, COL24A1, COL4A3, COL4A4, DDR2	CHRM2, KIF26B, KLC3, TACR1	none
ZnT10 treated	THSD7B, ADAMTS5	ADAMTSL4, GPC4, CSPG4, LUM, ADAMTS1, THBS2, FMOD	COL15A1, COL17A1, CTSV, F11R, FN1, HAPLN1, ITGA5, ITGA8, ITGB3, ITGB6, KDR, LAMB3, NID1, NID2, SPP1, THBS1	COL4A4, DDR2, LAMA2, MMP10, TTR	TACR1, CD4, SERPINA1, SYT2, CHRM2, APOA1, APOE, CTSC, EGFR, SH3KBP1	BTC, FOLR1, KIF19
ZIP14 treated	ADAMTSL1, ADAMTS16, THSD7B, MUC5B	SDC2, ADAMTS9, CSPG4, ADAMTS6	ADAM8, CDH1, CEACAM6, COL15A1, COL17A1, F11R, HAPLN1, ITGA2, ITGA8, KDR, LAMB3, MADCAM1, NID2, SPP1, TMPRSS6	COL24A1, COL4A4, DDR2, TTR, COL9A1, EMILIN3	TACR1, CD4, CTSC, HPX, SERPINA1, SYT2, VAMP8	COPZ2

Condition	Calcium Signalling pathway: Downregulated	Calcium Signalling pathway: Upregulated	Pathways of neurodegeneration: Downregulated	Pathways of neurodegeneration: Upregulated	Glucose metabolism: Downregulated	Glucose metabolism: Upregulated
ZIP14 basal condition	FLT1, ERBB3, KDR, EGF, CACNA1F, P2RX3, VEGFC, MET, ITPR3, NOS3, CACNA1B	FGF22, NTRK3, TACR3, TACR2, BDKRB2, LINC01238, ERBB4, P2RX7, DRD1, GRIN2A	MME, APOE, PPARG, CACNA1F, NOX4, NUP210L, PRPH, GPX7, ITPR3, NDUFA4L2, CACNA1B, MAP1LC3C	CACNA1B, MAP1LC3C, IRS1, LINC01238, BDNF, ERBB4, DNAI1, DNAH12, DNAI2, GRIN2A, SNCAIP	SLC2A3, SLC2A1, LDHA, PPP1R3C, ME1, ME3	LINC01238, ENO4, ALDH3B1
ZIP8 basal condition	FGF1, MYLK, PLCG2, TACR1, HTR2A, CHRM2, RYR2, CCKBR	HTR2C, NOS2, PLCD4, ITPKA	NDUFA4L2, SLC1A3, GPX7, PDYN, RYR2, GPX8, CSF1, EIF2AK2, GPX3	DNAH12, NOS2	SLC2A3, LDHA, PPP1R3C, ME1	None
ZnT10 basal condition	FLT1, KDR, ERBB3, FGF1, PDGFRA, PLCG2, ITPR3, PDGFRB, MYLK, RYR2	ITPKA, CHRNA7, NOS2, NOS1, NTRK1, MYLK2	MME, APOE, NDUFA4L2, ITPR3, WNT11, FAS, BACE2, GPX8, CSF1, CREB3L2, GPR37, PDYN, RYR2, MAPK13	SNCAIP, TUBA1C, CHRNA7, DNAI1, NOS2, NOS1, DNAH12	SLC2A3, PCK1, SLC2A1, LDHA, ME3, PPP1R3C	ENO4
Controls treated	FLT1, GRM5, GRPR, TACR1, RYR2, CHRM5, P2RX5, GRIN1, CAMK1G, CHRM2	PLCD4, MYLK3, FGF7, VEGFD, NOS2, DRD1	TGM2, GRM5, RYR2, CHRM5, GRIN1, KLC3, FZD8	NOS2, LPL	None	ENO4
ZnT10 treated	P2RX3, CHRNA7, P2RX5, CHRM3	FGFR2, ITPKB, CASQ2, PDGFRB, OXTR, ERBB3, PDGFRA, PDGFD, BDKRB2, CYSLTR1	CHRNA7, NEFL, NEFM, CHRM3, HAP1, SNCA	CREB5, HSPA5, DDIT3, BACE2, FAS, GPX8, WNT11, MME	PPP1R3C	PCK1
ZIP14 treated	NTRK1, GRIN2A, TACR2	ADORA2B, HTR2C, VEGFC, MET	DNAH11, SNCAIP, GRIN2A, DNAH12, DNAI1, NRG4, DNAH7, DNAH5	GPX7, TP53, FZD8, CSF1, APOE	None	PCK2, PPP1R3C, LDHA, SLC2A1

Condition	Glucose/Energy metabolism: Downregulated	Glucose/Energy metabolism: Upregulated	Respiratory electron transport, ATP synthesis by chemiosmotic coupling, and heat production by uncoupling proteins: Downregulated	Respiratory electron transport, ATP synthesis by chemiosmotic coupling, and heat production by uncoupling proteins: Upregulated	Dopaminergic neurogenesis and metabolism: Downregulated	Dopaminergic neurogenesis and metabolism: Upregulated
ZIP14 basal condition	CYP11A1, HNF4A, IGFBP3, IGFBP7, APOE, TK1, SLC2A1, PPARG, LDHA, NQO1, ALDH1A1, CA12, CEBPA, GAD2, TXNIP	IRS1, UCP3, LINC01238, PLIN1, NEUROD1, NKX6-1, CP	SLC2A1, PPARG, MMP9, LDHA, ATP6V1C2, TCIRG1, COL1A2, CEBPA, NDUFA4L2, NOS3	IRS1, UCP3, LINC01238, PLIN1	CDKN1C, ALDH1A1, GLI2, NQO1	LINC01238, SLC18A2, SHH, NR4A2, NEUROD1, LMX1B, NKX6-1, EN2
ZIP8 basal condition	CARTPT, CYP11A1, IGFBP7, CA2, LDHA, NQO1, ENPP1, YAP1, PANK1, TSPO, SLC4A4	None	LDHA, NDUFA4L2, RYR2	None	MAOA, MAOB, NQO1	None
ZnT10 basal condition	IGFBP3, IGFBP7, CYP11A1, APOE, PCK1, CA2, SLC2A1, ALDH1A1, CA12, LDHA, CEBPB, CEBPD, SGK1, SLC4A4, NQO1	STAR, NKX6-1	COL1A1, COL3A1, COL1A2, NDUFA4L2, MMP2, SLC2A1, ATP6V1C2, FAS, LDHA, CREB3L2, PDK4, RYR2, MAPK13	None	ALDH1A1, GLI2, NQO1	TH, NKX6-1
Controls treated	CYP11A1, CA12, SGK1	NEUROD1, ABCB1	RYR2	None	None	SHH, NEUROD1
ZnT10 treated	None	MTHFD2, PHGDH, CEBPB, ATF3, IGFBP7, MB, ABCB1, PCK1, ALDH1A1	None	SUCLG2, CREB5, DDIT3, MMP2, FAS, JUN, TGFB2, SOCS3, COL1A2, COL3A1, COL1A1	None	ASCL1, SLC18A2, ALDH1A1
ZIP14 treated	NKX6-1, FOXA1	MTHFD2, SLC1A5, ATF3, IP6K3, PCK2, CTH, LDHA, NQO1, SLC2A1, PCSK9, CA12, APOE, ALDH1A1, TYR	CD36	LDHA, SLC2A1	NKX6-1	GLI2, ALDH1A1

Shared between 2 disorders
Shared between all disorders

Table S4. Shared DEGs associated with SLC39A14, SLC39A8, and SLC30A10 disorders using MitoCarta 3.0, related to Figure 2.

ZIP14, basal condition		ZIP8, basal condition		ZnT10, basal condition	
**Downregulated genes**					
CYP11A1	Metabolism<br>Lipid metabolism<br>Metals and cofactors<br>Heme-containing proteins	CYP11A1	Metabolism<br>Lipid metabolism<br>Metals and cofactors<br>Heme-containing proteins	CYP11A1	Metabolism<br>Lipid metabolism<br>Metals and cofactors<br>Heme-containing proteins
IFI27	signaling<br>Mitochondrial dynamics and surveillance<br>Apoptosis	IFI27	signaling<br>Mitochondrial dynamics and surveillance<br>Apoptosis	IFI27	signaling<br>Mitochondrial dynamics and surveillance<br>Apoptosis
STOM	Protein import, sorting and homeostasis	ECHDC2	Unknown	STOM	Protein import, sorting and homeostasis
CKMT2	Metabolism<br>Nucleotide metabolism<br>Creatine metabolism	CKMT2	Metabolism<br>Nucleotide metabolism<br>Creatine metabolism	CKMT2	Metabolism<br>Nucleotide metabolism<br>Creatine metabolism
ACCS	unknown	ACCS	unknown	ACCS	unknown
MGST1	Metabolism<br>Detoxification<br>ROS and glutathione metabolism	DNAJC15	Protein import, sorting and homeostasis<br>Import motor	DNAJC15	Protein import, sorting and homeostasis<br>Import motor
PRSS35	Protein import, sorting and homeostasis	MAOB	Metabolism<br>Amino acid metabolism<br>Catechol metabolism<br>Detoxification	PMAIP1	Mitochondrial dynamics and surveillance<br>Apoptosis
PMAIP1	Mitochondrial dynamics and surveillance<br>Apoptosis	MAOA	Metabolism<br>Amino acid metabolism<br>Catechol metabolism<br>Detoxification	HTATIP2	unknown
HTATIP2	unknown	DARS2	Mitochondrial central dogma<br>Translation<br>mt-tRNA synthetases	PRSS35	Protein import, sorting and homeostasis
ACOT2	Metabolism<br>Lipid metabolism	TSPO	Metabolism<br>Lipid metabolism<br>Cholesterol, bile acid, steroid synthesis	ECHDC3	unknown
ME3	Metabolism<br>Carbohydrate metabolism<br>TCA<br>Nucleotide metabolism<br>Nucleotide synthesis and processing	TRMT2B	Mitochondrial central dogma<br>mtRNA metabolism<br>mtRNA modifications	ECHDC2	unknown
		EHHADH	Metabolism<br>Lipid metabolism	CPS1	Metabolism<br>Amino acid metabolism<br>Urea cycle
		MSRB3	Metabolism<br>Detoxification<br>ROS and glutathione metabolism	ACSF2	Metabolism<br>Lipid metabolism<br>Fatty acid oxidation
				ME3	Metabolism<br>Carbohydrate metabolism<br>TCA<br>Nucleotide metabolism<br>Nucleotide synthesis and processing
				PDK4	Metabolism<br>Carbohydrates metabolism<br>Pyruvate metabolism
				DARS2	Mitochondrial central dogma<br>Translation<br>mt-tRNA synthetases
				SLC25A4	Small molecule transport
**Upregulated genes**					
UCP3<br>ABCD2		None		STAR	

Shared genes

ZIP14 TR vs UT		ZnT10 TR vs UT		Control TR vs UT	
**Upregulated genes in Treated patients or control**					
IFI27	signaling<br>Mitochondrial dynamics and surveillance<br>Apoptosis	MTHFD2	Metabolism<br>Vitamin metabolim<br>Folate and 1-C metabolism	SQOR	Mitochondrial central dogma, mt-tRNA synthetases<br>Metabolism<br>Electron carriers<br>Sulfur metabolism
SLC25A4	Small molecule transport	STOM	Protein import, sorting and homeostasis	MAOB	Metabolism<br>Amino acid metabolism<br>Catechol metabolism<br>Detoxification
STOM	Protein import, sorting and homeostasis	CYP27A1	Metabolism<br>Lipid metabolism<br>Metals and cofactor<br>Heme-containing proteins		
PCK2	Metabolism<br>Carbohydrates metabolism<br>Gluconeogeneis	SUCLG2	Metabolism<br>Carbohydrates metabolism<br>TCA cycle<br>Nucleotide metabolism<br>Nucleotide synthesis and processing		
MGST1	Metabolism<br>Detoxification<br>ROS and glutathione metabolism	ECHDC3	Unknown		
MPV17L	Mitochondrial central dogma<br>Translation<br>Mitochondrial ribosome assembly<br>Small molecule transport	FKBP10	Protein import, sorting and homeostasis<br>Protein homeostasis<br>Chaperones		
FKBP10	Protein import, sorting and homeostasis<br>Chaperones	PRSS35	Protein import, sorting and homeostasis		
MTHFD2	Metabolism<br>Vitamin metabolim<br>Folate and 1-C metabolism				
DMGDH	Metabolism<br>Amino acid metabolism<br>Glycine metabolism<br>Vitamin metabolim<br>Choline and betaine metabolism<br>Folate and 1-C metabolism				
**Downregulated genes in Treated patients or control**					
BCO2		None		CKMT2<br>CYP11A1	

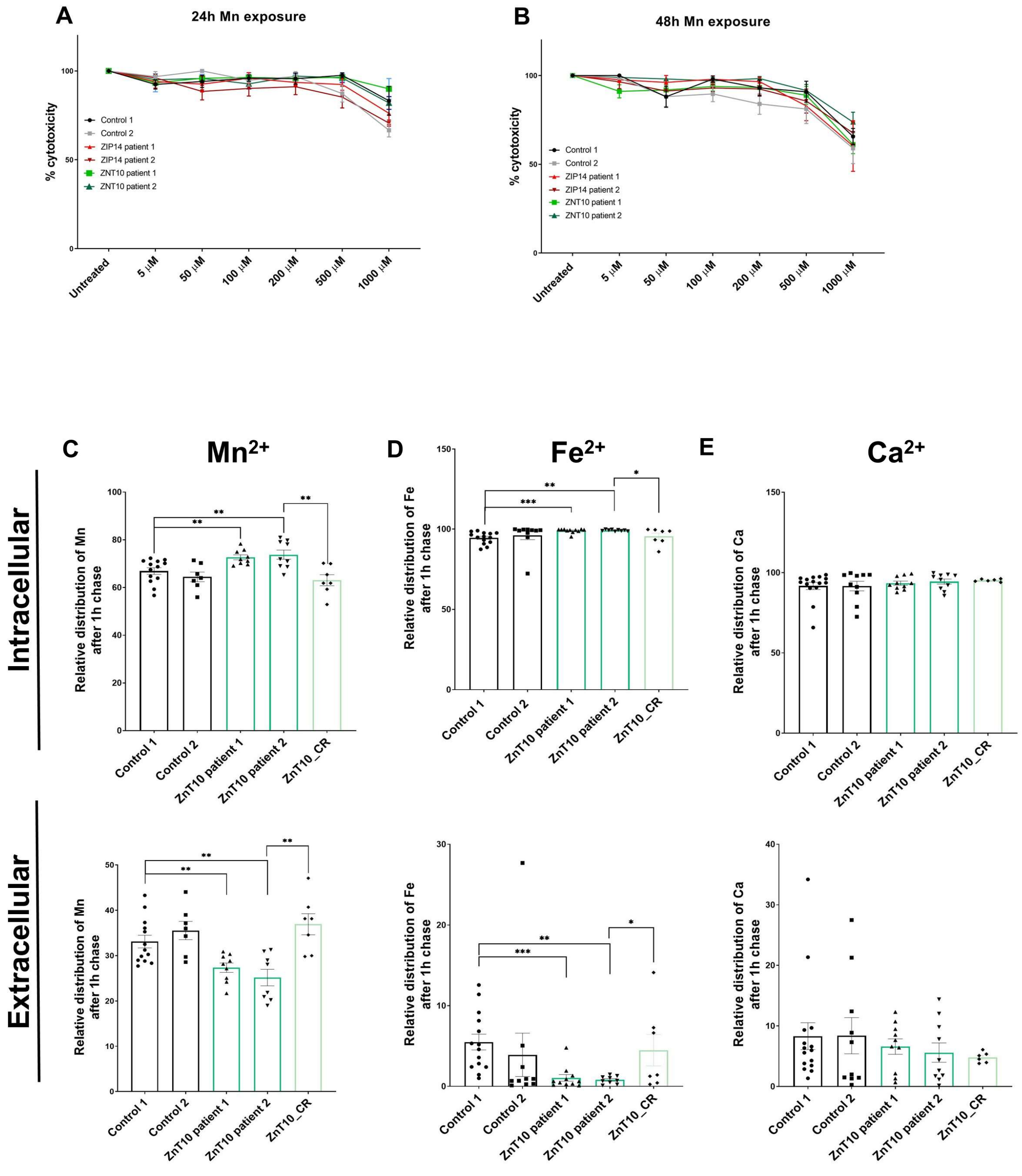

Figure S6. Pulse chase assay confirms manganese efflux activity of ZnT10, related to Figure 3.

(A-B) MTT assay confirms that exposure to 100 μm $MnCl_2$ for 48h does not cause significant cell death in mDA neurons.

(C-E) Relative distribution of manganese (D), iron (E), calcium (F) between intracellular and extracellular compartments. n= 6 – 15 biological replicates, unpaired Student's t test; *p=0.05-0.01, **p=0.01-0.001, p***< 0.001. Values are given as means ± SEM.

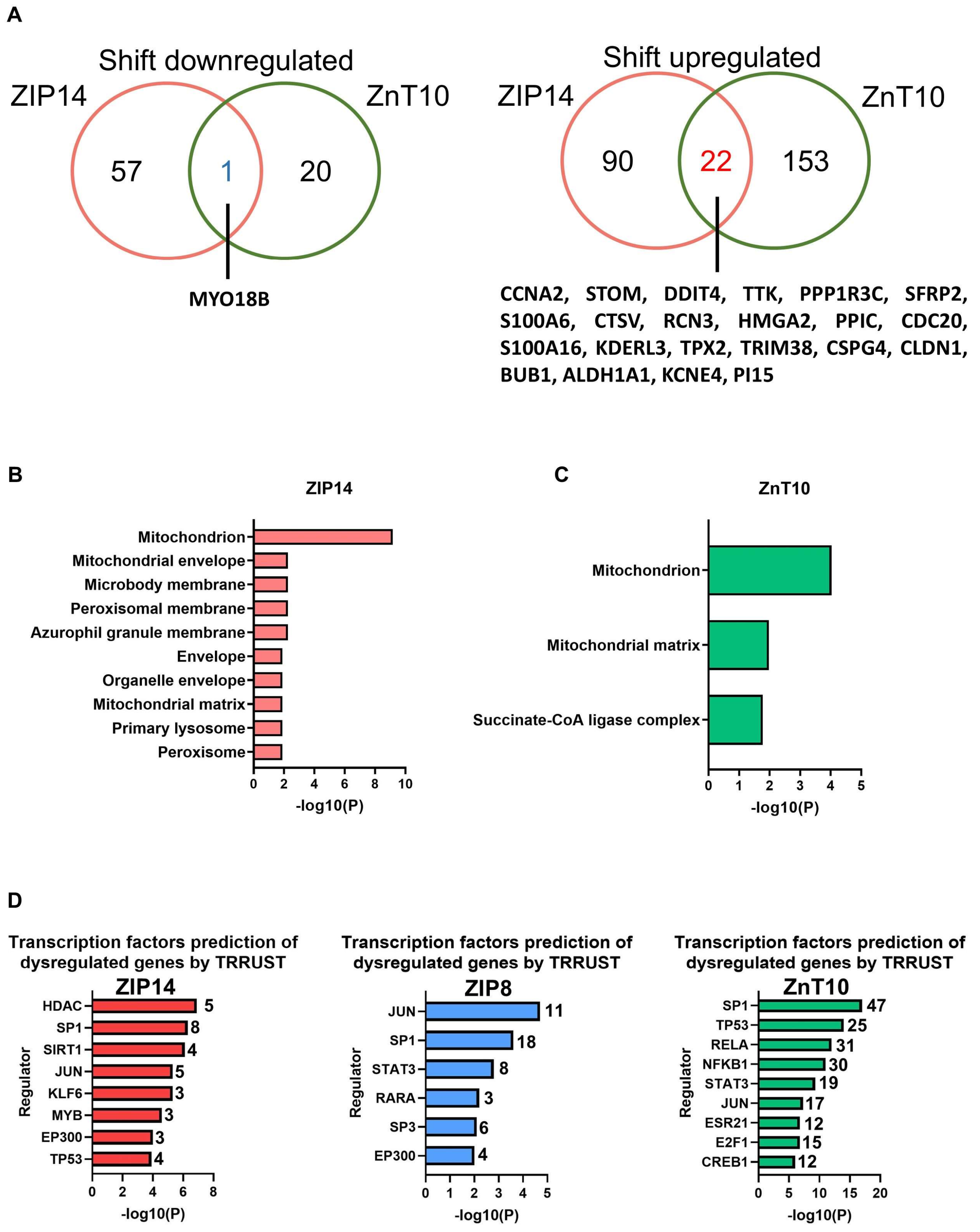

Figure S7. Mn-driven transcriptional effects on patient lines, related to Figure 3 and Figure 4.

(A) Venn diagrams illustrating genes whose expression is reversed by Mn treatment, highlighting shifts from downregulation to upregulation and vice versa.

(B-C) Gene ontology (GO) terms enrichment for cellular process dysregulated in ZIP14 (B) and ZnT10 (C) patient lines upon Mn exposure. These terms were highlighted by cross-referencing DEGs with the MitoCarta 3.0 database and enrichment was performed using ShinyGO 0.76.2.

(D) Transcription factors revealed by TRRUST analysis in patient lines. Top transcription factors (-log10(P)) of dysregulated transcript in ZIP14, ZIP8, and ZnT10 mDA neurons compared to control lines. The number of dysregulated genes they regulate is adjacently indicated.

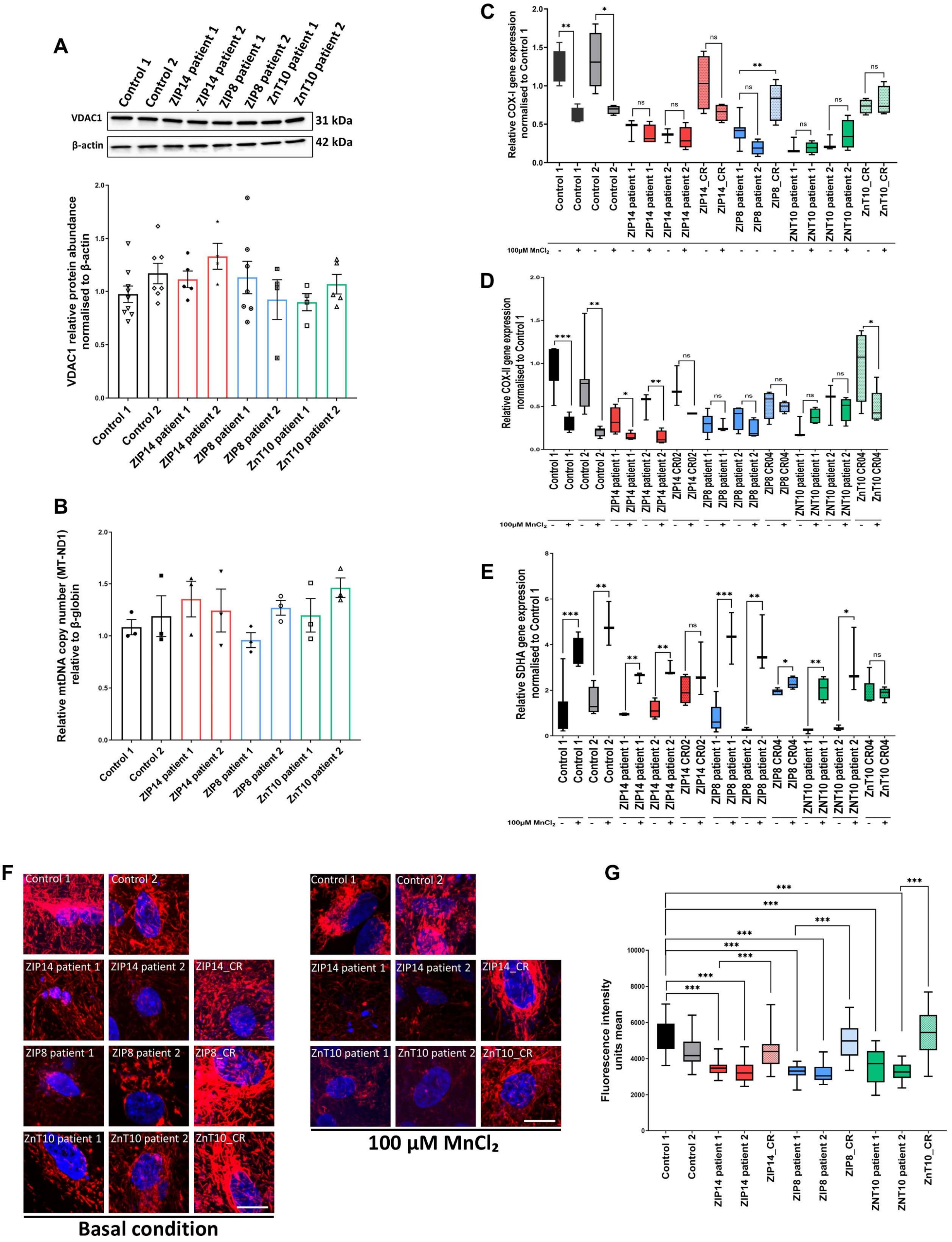

Figure S8. Mitochondrial integrity and gene expression, related to Figure 5.

(A) Immunoblot analysis for VDAC1 (A) and relative quantification (B) shows no difference in mitochondrial mass between lines. n= 3 – 7 biological replicates, unpaired Student's t test; *p=0.05-0.01, **p=0.01-0.001, p***< 0.001. Values are given as means ± SEM.

(B) qRT-PCR analysis for mtDNA copy number of mitochondrial gene MT-ND1, normalized to β-globin. n= 3 biological replicates, unpaired Student's t test; *p=0.05-0.01, **p=0.01-0.001, p***< 0.001. Values are given as means ± SEM.

(C-E) qRT-PCR analyses for COX-I (C), COX-II (D), and SDHA (E), relative to GAPDH and normalized to an internal control. n= 3 – 7 (COX-I), n=3 – 7 (COX-II), n=3 – 14 (SDHA) biological replicates, unpaired Student's t test; *p=0.05-0.01, **p=0.01-0.001, p***< 0.001. Box-and-whisker plot shows median with min to max values. Values are given as means ± SEM.

(F) Representative zoomed-in TMRM immunostaining in mature neurons, in both physiological and manganese exposed conditions. Scale bar = 30 µm.

(G) TMRM fluorescence intensity measurements in basal condition, between control and patient lines. n= 3 – 4 biological replicates, N=3 – 12 technical replicates (fields of view), unpaired Student's t test; *p=0.05-0.01, **p=0.01-0.001, p***< 0.001. Box-and-whisker plot shows median with min to max values. Values are given as means ± SEM.

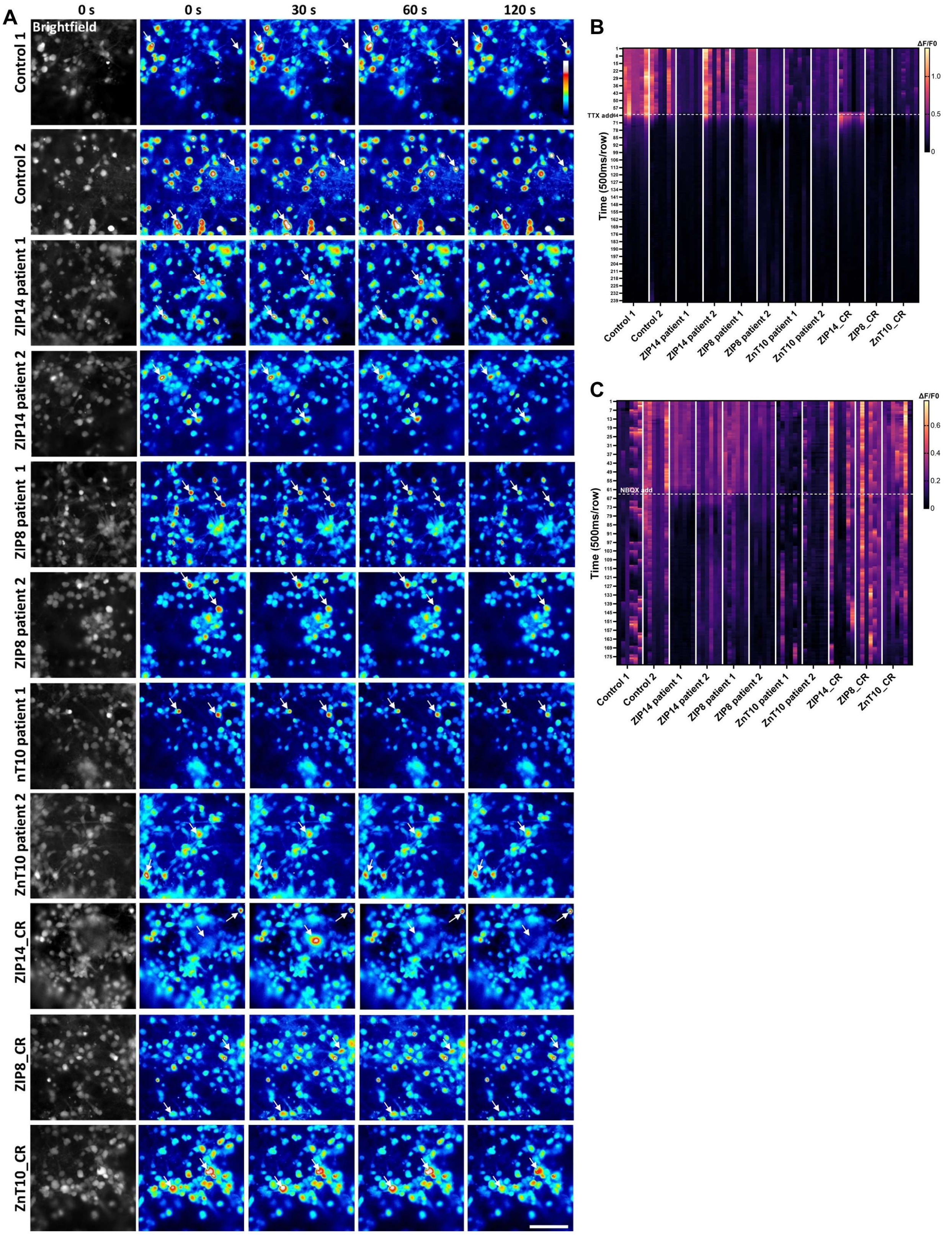

Figure S9. Measurement of calcium signalling in control and patient lines, and following addition of modulators of neuronal excitability, related to Figure 7.

(A) Representative Ca^{2+} transients over a 2 min period in neurons labelled with Calbryte 520 AM. Arrows indicate cells with change in Ca^{2+} fluxes over time (red to blue: high to low fluorescence intensity),

(B) Ca^{2+} transients measured following addition of 10 μM TTX, for a total of 120 s. Scale represents ΔF/F0 fluorescence intensity, ranging from 0 (low ΔF/F0, dark purple) to 0.8 (high ΔF/F0, yellow). n=6 representative cells in one experiment.

(C) Ca^{2+} transients measured following addition of 10 μM NBQX, for a total of 120 s. Scale represents ΔF/F0 fluorescence intensity, ranging from 0 (low ΔF/F0, dark purple) to 0.8 (high ΔF/F0, yellow). n=6 representative cells in one experiment.
